# A_2B_ Adenosine Receptor and Cancer

**DOI:** 10.3390/ijms20205139

**Published:** 2019-10-17

**Authors:** Zhan-Guo Gao, Kenneth A. Jacobson

**Affiliations:** Molecular Recognition Section, Laboratory of Bioorganic Chemistry, National Institute of Diabetes and Digestive and Kidney Diseases, National Institutes of Health, Bethesda, MD 20892-0810, USA

**Keywords:** adenosine receptor, immune system, cancer therapy, tumor microenvironment, cell proliferation, metastasis

## Abstract

There are four subtypes of adenosine receptors (ARs), named A_1_, A_2A_, A_2B_ and A_3_, all of which are G protein-coupled receptors (GPCRs). Locally produced adenosine is a suppressant in anti-tumor immune surveillance. The A_2B_AR, coupled to both Gαs and Gαi G proteins, is one of the several GPCRs that are expressed in a significantly higher level in certain cancer tissues, in comparison to adjacent normal tissues. There is growing evidence that the A_2B_AR plays an important role in tumor cell proliferation, angiogenesis, metastasis, and immune suppression. Thus, A_2B_AR antagonists are novel, potentially attractive anticancer agents. Several antagonists targeting A_2B_AR are currently in clinical trials for various types of cancers. In this review, we first describe the signaling, agonists, and antagonists of the A_2B_AR. We further discuss the role of the A_2B_AR in the progression of various cancers, and the rationale of using A_2B_AR antagonists in cancer therapy.

## 1. Introduction

**Figure 1:**
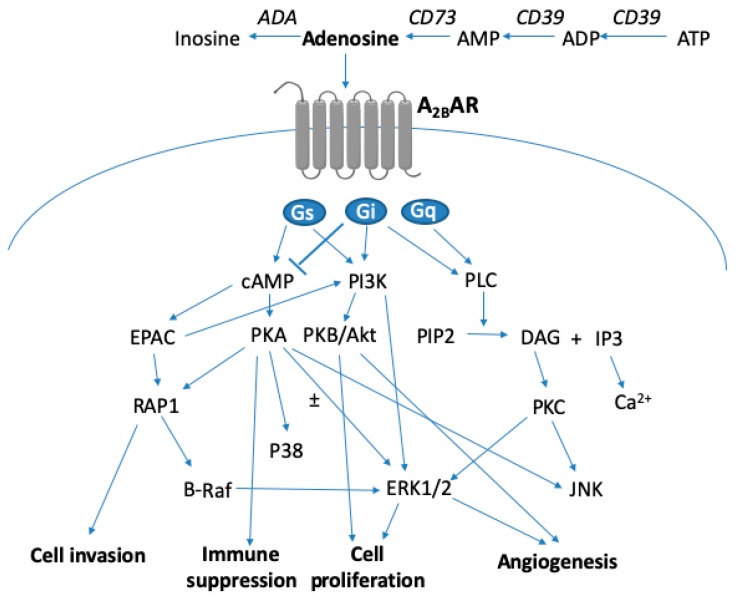
A_2B_ adenosine receptor (AR) signaling in mammalian cells and in the tumor microenvironment, as explained in the text. The three G proteins shown act through either G α, e.g., on cyclic AMP (cAMP), or G β, γ subunits, e.g., on phosphoinositide 3-kinase (PI3K). Protein kinase A (PKA) has either a stimulatory or inhibitory effect on extracellular signal-regulated kinase 1/2 (ERK1/2). For more detail see: [15,35,49,56,58]. For effects on specific immune cells, see [17,32].

Adenosine, in the extracellular milieu, is generated mainly via the degradation of adenosine 5′-triphosphate (ATP) released under stress conditions, to protect cells and tissues locally. Adenosine and ATP acting at different classes of receptors often have opposite effects in cell proliferation or cell death. ATP and other adenine nucleotides have antitumor effects via the activation of the P2Y_1_ receptor (P2Y_1_R) subtype [1,2], whereas adenosine induces cancer cell proliferation and growth of many types of tumors via the activation of the A_2B_ adenosine receptor (AR) [3,4,5,6,7,8,9,10]. The generation and degradation/removal of adenosine is a multi-step and balanced process in cells involving enzymes (Cluster of Differentiation 39 (CD39), CD73, CD26, adenosine deaminase, adenosine kinase, S-adenosyl homocysteine hydrolase) and nucleoside transporters [11], which are not the main topic of this review. Although extracellular adenosine exerts its action via four G protein-coupled receptors (GPCRs), A_1_, A_2A_, A_2B_ and A_3_ [12], in this review we will only focus on the importance of A_2B_AR signaling (Figure 1) in cancer progression and the rationale to use A_2B_AR antagonists as anticancer agents.

**Figure 2:**
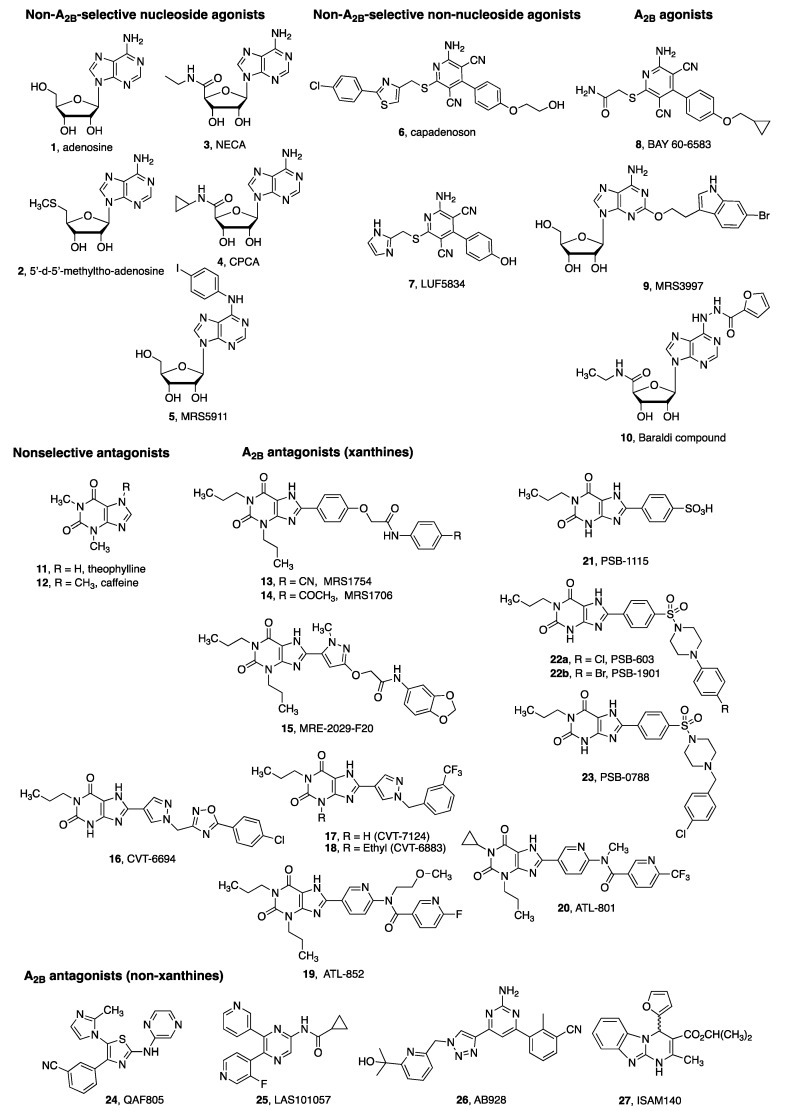
Chemical structures of both commercially available and literature-reported A_2B_AR agonists (1–10) and antagonists (11–27) as pharmacological tools and an A_2A_AR/A_2B_AR mixed antagonist (26) in a clinical trial for cancer treatment. For more detail, see [36,37].

The importance of the A_2B_AR in cancer progression has only recently been revealed, despite the physiological role of adenosine in cardiac function being realized almost a century ago [13]. Although A_2B_AR effects in brain slices were characterized in the early 1980s [14], until recently the A_2B_AR has been poorly characterized in comparison to the other three ARs, which is at least in part due to the fact that A_2B_AR has low affinity for the endogenous agonist adenosine 1 (EC_50_ ~24 µM, Figure 2, Table 1). Thus, it was assumed that A_2B_AR must have a minor physiological significance. However, increasing evidence has shown that there is a dramatic increase in extracellular adenosine concentration and a significant upregulation of A_2B_AR expression under many pathological conditions [15,16,17], such as hypoxia, inflammation and cancer, which may indicate the critical role of A_2B_AR in many diseases. For example, adenosine concentration has been reported to increase 10-fold in patients with septic shock [18]. Hypoxia-inducible factor 1 (HIF-1α) has been reported to up-regulate A_2B_AR expression on activated macrophages [19]. Lan et al. [20] found that hypoxia increased expression of A_2B_AR in human breast cancer cells through the transcriptional activity of HIF-1α. The discovery that A_2B_AR expression is significantly increased by HIF-1α strongly suggested its involvement in cancer promotion [20,21,22,23]. In addition to its role in tumor growth, inhibition of A_2B_AR genetically or pharmacologically dramatically decreased lung metastasis after implantation of breast cancer cells into the mammary fat pad of immunodeficient mice [20]. It has also been recently shown that bladder urothelial carcinoma expresses high levels of A_2B_AR, which is suggested to be associated with a poor patient prognosis [24]. A tissue microarray of 232 breast cancer samples, that included 66 triple negative breast cancer cases suggest that A_2B_AR could serve as a prognostic biomarker and a potential therapeutic target [25]. Kasama et al. [6] showed that A_2B_AR controls cellular proliferation via HIF-1α activation, indicating that A_2B_AR may be a key regulator of tumoral progression in oral squamous cell carcinoma. Thus, the A_2B_AR is consistently and convincingly demonstrated to be involved in tumor cell proliferation, metastasis, angiogenesis, and immune suppression. Furthermore, the A_2B_AR and A_3_AR seem to be the only AR subtypes that are expressed in significantly higher levels in cancer tissues in comparison to normal adjacent tissues, similar to several other GPCRs [6,8,26,27,28].

Although all four ARs are reported to be involved in cancer progression [16,29,30,31], the other three ARs have been shown both to be pro- and anti-tumoral [16]. For example, both pro- and anti-tumoral effects have been reported for the A_1_AR [16]. Targeting A_2A_AR has been considered a double-edged sword [16,32]. It has been suggested that adenosine accumulation in the tumor microenvironment facilitates tumor growth through the inhibition of effector T cells and natural killer (NK) cells [33], and inhibition of A_2A_AR alone was found to be sufficient to establish anti-tumor immunity and protect against metastasis in various mouse models of cancer [33]. However, A_2A_AR deletion does not inhibit the growth of all tumor types and might have the opposite effect. For example, an increased tumor growth rate of both B16F10 melanoma and MB49 bladder carcinomas has been observed in A_2A_AR knockout (KO) mice [5]. Blocking A_2B_AR action might have advantages over the A_2A_AR as a cancer therapeutic target. Cekic et al. [5] showed that AR antagonist theophylline slowed the growth of MB49 bladder and 4T1 breast tumors in mice and reduced breast cancer cell metastasis from mammary fat to the lung via the A_2B_AR, but not the A_2A_AR, based on experiments using A_2A_AR or A_2B_AR KO mice. The role of A_3_AR has been investigated in various cancer cell types with contrasting results, i.e., both pro- and anti-proliferative, as well as pro-apoptotic and anti-apoptotic effects [15]. Both A_3_AR agonists and antagonists have been considered for anti-cancer agents, although only A_3_AR agonists have progressed in clinical trials [28].

Recent advances in the signaling and function of the A_2B_AR [17,32,34,35] and the availability of selective ligands [36,37,38], have greatly facilitated understanding of the role of A_2B_AR in cancer progression and the rationale for the development of A_2B_AR antagonists as anti-tumor drugs. In this review, we first describe the distribution, signaling, agonists, and antagonists of the A_2B_AR. We then discuss the role of the A_2B_AR in the progression of various types of cancers, and the rationale of using A_2B_AR antagonists in cancer therapy.

## 2. A_2B_AR Distribution and Expression

In rats, A_2B_AR mRNA was detected at various levels in all tissues studied [39]. In mice, by replacing exon 1 of the A_2B_ gene with a reporter construct containing β-gal, mouse tissue-specific activation of the A_2B_ gene promoter was conveniently determined in various organs and specific cells within those organs, with the primary site of expression being the vasculature [40]. Yang et al. [40] found that mouse smooth muscle cells, endothelial cells and macrophages exhibit high A_2B_AR expression levels. The high level of A_2B_AR expression in endothelial cells suggests a potential role in angiogenesis. In human primary cells, A_2B_AR has been found in endothelial cells, mast cells, dendritic cells, macrophages, and neutrophils [17,41]. High expression levels in dendritic cells and macrophages indicate a possible role in modulation of immunity. In human cancer tissues, A_2B_AR expression levels were found to be higher than in adjacent normal tissues [6,8,24,26,27]. High levels of A_2B_AR have been suggested to be associated with worse prognosis in bladder urothelial carcinoma [24]. Mittal et al. [7] suggested that high A_2B_AR expression is associated with worse prognoses in triple-negative breast cancer (TNBC). In a TNBC mouse model, A_2B_AR activation increased metastasis [42], while A_2B_AR antagonism in mouse models reduced the tumor burden by immune mechanisms and action on tumor cells. The high A_2B_AR expression has also been found in many human tumor cell lines, such as PC3 prostate, T24 bladder, 1321N1 astrocytoma [35], U373MG astrocytoma [43], MDA-MB-231 breast [20], Jurkat T cells [44], BON-1 pancreatic and KRJ-I intestinal [45], A375 melanoma [46], and THP-1 human monocytes [47]. The high expression of the A_2B_AR in those cancer cells indicates its potential role in cancer progression. In a glioblastoma cell line derived from a mouse line containing spatially expressed A_2B_AR, this receptor is highly upregulated leading to proliferation, angiogenesis and invasiveness [48]. Mouse KO of CD73, which forms adenosine locally from adenosine 5′-monophosphate (AMP), reduced A_2B_AR signaling in the glioblastoma, to decrease pathogenesis and increase sensitivity to chemotherapy. A_2B_AR expression was also demonstrated in human lung epithelial cells [49]. Consistent with the high A_2B_AR expression in bladder cancer and breast cancer cells, Cekic et al. [5] showed that A_2B_AR antagonists delayed the growth of bladder and breast tumors and reduced lung metastasis. Lan et al. [20] found that genetic or pharmacological inhibition of A_2B_AR expression or activity dramatically impaired tumor initiation and lung metastasis in mice. Thus, high A_2B_AR expression is related to tumor growth and metastasis, and therefore A_2B_AR antagonists are potential therapeutic agents for various types of cancers including lung cancer.

## 3. A_2B_AR Signaling

Classical A_2B_AR signaling has been initially and primarily demonstrated in Chinese hamster ovary (CHO) cells expressing the recombinant human A_2B_AR [50,51,52]. A_2B_AR activation leads to dissociation of the Gαs and Gβγ subunits and subsequent activation of the adenylyl cyclases, which in turn hydrolyze intracellular ATP into cyclic AMP (cAMP), which activates protein kinase A (PKA) and many downstream signaling molecules. The Gs-cAMP-PKA axis is an important A_2B_AR-mediated signaling pathway. For example, Xu et al. [53] found that A_2B_AR-mediated cAMP is both necessary and sufficient to suppress interferon-γ (IFN-γ)-mediated immune responses. Jing et al. [54] showed that A_2B_AR activation in hematopoietic stem cells induced chemokine (C-X-C motif) ligand 8 (CXCL8, interleukin 8) production via cAMP-PKA signaling and mediated hematopoiesis. In addition to PKA, cAMP also activates ‘exchange protein directly activated by cAMP’ (EPAC), another important signaling molecule related to cell migration and angiogenesis [55]. In CHO cells expressing the recombinant human A_2B_AR, the nonselective AR agonist NECA 3 activated cAMP response element-binding protein (CREB) and P38 (a mitogen-activated protein kinase, MAPK) but not Akt (protein kinase B). Extracellular signal-regulated kinase 1/2 (ERK1/2) and GTPase Rap1 were blocked by PKA inhibitor H89 [52]. Phosphorylation of Akt and ERK1/2 was blocked by a phosphoinositide 3-kinase (PI3K) inhibitor, wortmannin. Thus, A_2B_AR activating various downstream MAPKs may be via different signaling pathways. Although PKA-independent in CHO cells, Rap1 activation seems PKA-dependent in human embryonic kidney (HEK)293 cells [56]. The coupling of A_2B_AR to β-arrestin signaling has also been reported [37,57].

Most of the early studies on A_2B_AR signaling utilized CHO or HEK293 cells transfected with recombinant human A_2B_AR [51,52]. However, in various types of cells endogenously expressing A_2B_AR, the receptor was able to couple to either Gi or Gs, depending on the cell type and downstream signaling pathway measured [35]. For example, A_2B_AR agonist NECA stimulates ERK1/2 phosphorylation via Gαi in T24 bladder cancer cells [35], but via Gαs in CHO cells [52]. The Gαi inhibitor pertussis toxin, but not Gαq KO, diminished NECA-stimulated ERK1/2 activity suggesting the involvement of Gαi rather than Gαq [35]. A_2B_AR downregulates ERK1/2 activity via Gαs in 1321N1 astrocytoma cells [35] and in MDA-MB-231 breast cancer cells [58]. ERK1/2 reduction in MDA-MB-231 cells was triggered by an A_2B_AR agonist and forskolin, but abolished by the PKA inhibitor H89, suggesting an important role for the cAMP-PKA pathway in controlling ERK1/2 activity in MDA-MB-231 cells. A_2B_AR-mediated intracellular calcium mobilization in T24 cells was mainly via Gi, although Gs may also play a minor role, but Gq is not involved [35]. Thus, it is conceivable that in many cases the predominant A_2B_AR coupling is through Gαi rather than Gαs. Many important A_2B_AR functions from primary cells or tissues have recently been related to the PI3K-Akt and Ras-related protein (RAP)1B-EPAC pathways [59,60,61,62,63]. However, it has not been extensively explored whether those signaling molecules are actually downstream of A_2B_AR-mediated Gαi or Gαs proteins. The A_2B_AR-mediated major signaling pathways are illustrated in Figure 1.

## 4. A_2B_AR Agonists and Antagonists as Pharmacological Tools

Although numerous antagonists and a few agonists for the A_2B_AR have been reported, in this section we focus on the agonists and antagonists that are commercially available as pharmacological tools and those in clinical trials for cancer patients (Table 1). In addition to selective antagonists and agonists, various specialized pharmacological tools can be used to characterize A_2B_AR and its activity. Radiolabelled compounds are used to investigate A_2B_AR binding activity including both tritiated ligands and ^18^F-labeled compounds for positron emission tomography [64,65]. Ligands that have been tritiated for A_2B_AR binding experiments are: agonists 3 and 8; antagonists 13, 21, 22a, and ZM241,385 (structure not shown). Fluorescent antagonists of high affinity at the A_2B_AR were recently reported [66,67]. A_2B_AR allosteric modulators have been reported but not extensively characterized [36].

There are two major classes of A_2B_AR agonists that are commercially available (Figure 2). The adenosine derivatives include adenosine, NECA and CPCA 4, which are considered as full and balanced agonists and often used as standard A_2B_AR agonists albeit nonselective [37,68]. The non-adenosine 3,5-dicyanopyridine class of A_2B_AR agonists that are commercially available include BAY60-6583 8, LUF5834 **7** and BAY68-4986 (A_1_AR agonist Capadenoson 6). BAY60-6583 is an A_2B_AR-selective agonist but shows variable agonist E_max_ and potencies in different types of cells and tissues ([35,37]. Partial and biased agonists for the A_2B_AR have been reported [34,35,37,69,70]. In cAMP accumulation assays, 5′-substituted nucleosides NECA and CPCA, and non-adenosine agonists BAY60-6583 and BAY68-4986 are all full agonists in cells overexpressing the recombinant human A_2B_AR. In calcium mobilization, ERK1/2 phosphorylation and β-arrestin translocation, only 5′-substituted adenosine analogs CPCA and NECA are full agonists. A quantitative operational model characterized BAY60-6583 as an ERK1/2-biased agonist and N⁶-substituted agonists as biased against calcium and β-arrestin pathways. Interestingly, a partial A_2B_AR agonist BAY60-6583 behaved as an A_2B_AR antagonist in MIN-6 mouse pancreatic β cells expressing low A_2B_AR levels, to induce insulin release [37]. It remains to be determined whether BAY60-6583 behaves as a partial agonist or an antagonist in other cell types endogenously expressing low levels of the A_2B_AR.

A_2B_AR expression levels often determine the potency and E_max_ of a given A_2B_AR agonist. BAY60-6583 was found to be a partial agonist in stimulating cAMP accumulation in several cell types endogenously expressing the A_2B_AR [37]. For example, in an assay of cAMP accumulation in HEK293 cells endogenously expressing the A_2B_AR, the EC_50_ and agonist E_max_ values of BAY60-6583 are 242 nM and 73%, respectively. However, in HEK293 cells overexpressing the recombinant A_2B_AR, the EC_50_ and E_max_ of BAY60-6583 are 6.1 nM and 102%, respectively [37]. BAY60-6583 did not show any agonist activity in stimulating calcium mobilization or ERK1/2 phosphorylation in T24 bladder cancer cells. BAY60-6583 also did not show any agonist activity in stimulating calcium transients in HEK293 cells, although it showed a robust effect in stimulating cAMP accumulation and ERK1/2 activity. LUF5834 has been reported as a nonselective A_2B_AR agonist showing an EC_50_ of 12 nM in cAMP accumulation and an agonist E_max_ of 74% in comparison with NECA (E_max_ = 100%) [71]. Using CHO cells overexpressing the human A_2B_AR, Baltos et al. (2017) [70] found that the A_1_AR agonist BAY68-4986 shows potent A_2B_AR agonist activity in stimulating cAMP accumulation, with an EC_50_ of 1.1 nM. However, when tested in cAMP accumulation in HEK293 cells endogenously expressing the A_2B_AR, BAY68-4986 showed an EC_50_ of 500 nM and E_max_ of 95% (Gao and Jacobson, unpublished data). Thus, for all nucleoside and non-nucleoside A_2B_AR agonists commercially available, only the partial agonist BAY60-6583 is A_2B_AR selective, which may show agonist activity in some signaling pathways, and antagonist properties in other signaling events [37]. Full agonists selective for A_2B_AR are not yet available. Future efforts could be the development of selective and full agonists for A_2B_AR, in order to have a full range of A_2B_AR efficacies for studying cell proliferation, angiogenesis, metastasis and immune suppression.

The structures and potencies of the commercially available antagonists as pharmacological tools are listed in Figure 2 and Table 1, respectively. The first selective A_2B_AR antagonists were reported by [72], which were xanthine derivatives, and currently there are chemically diverse heterocyclic selective A_2B_AR antagonists, such as recently reported LAS101053 25, AB928 26 and ISAM140 27 [3,36]. Commercially available A_2B_AR antagonists as pharmacological tools include 8-arylxanthine derivatives MRS1754 13, MRS1706 14, GS6201 18, PSB-1115 21, PSB-603 22a and PSB-0788 23. Recently, an alkylxanthine with a picomolar affinity at the human A_2B_AR, PSB-1901 22b, was reported [73]. Antagonists that are in clinical trials (AB928 26, PBF-1129 and theophylline 11) will be discussed in Section 9.

## 5. A_2B_AR in Cell Proliferation and Tumor Growth

A_2B_AR activation can promote proliferation of multiple types of cancer cells and growth of solid tumors. Activation of the A_2B_AR by BAY 60–6583 was shown to stimulate both proliferation and migration of MDA-MB-231 cells [78]. The A_2B_AR-mediated effects were blocked by an A_2B_AR antagonist GS-6201. Wei et al. [1] found the A_2B_AR to be the most highly expressed AR in several human prostate cancer cell lines, including PC-3, and A_2B_AR activation promotes cell proliferation and decreases cell apoptosis. An A_2B_AR-selective antagonist PSB-603 decreased proliferation of prostate cancer cell lines [4,79], and colon cancer cells [21]. Activation of the A_2B_AR with agonist BAY 60-6583 increased tumor growth in a mouse model of melanoma [10]. In a model of bladder cancer, inhibition of tumor growth by the non-selective antagonist theophylline was demonstrated to be mediated by A_2B_AR but not A_2A_AR blockade [5]. A_2B_AR selective antagonist ATL801 also inhibited growth of MB49 bladder and 4T1 breast tumor volume [5] and melanoma in mice [10]. Stagg et al. [80] showed that A_2B_AR activation promoted 4T1.2 tumor-cell chemotaxis in vitro and metastasis in vivo. High A_2B_AR expression levels have also been found in hepatocellular carcinoma [27]. It has been suggested that high A_2B_AR levels are generally associated with worse prognosis or poor survival [7,17].

The results from A_2B_AR blockade with antagonists were consistent with those from genetic knockdown and KO of the A_2B_AR in various animal models of solid tumors [5,6,9], further confirming the critical role of this receptor in cancer cell proliferation and growth.

The specific mechanisms related to A_2B_AR-mediated proliferation of various cancer cells and growth of different types of tumors have not been extensively and systematically explored. As it has been suggested that different agonists may bind in different modes and induce different A_2B_AR conformational changes [81], together with the recent finding that A_2B_AR may couple variably to at least three G proteins in different cell types, it is possible that each agonist may activate a particular mix of signaling cascades in a specific cell type, or the same agonist may activate different signaling pathways in other cell types [35]. Thus, the signaling mechanisms related to A_2B_AR-mediated cell proliferation may be diverse in different types of cancers. Nevertheless, multiple studies have shown the importance of several signaling pathways related to A_2B_AR activation and the subsequent release of various cytokines and growth factors, which eventually led to cancer cell proliferation. MAPK signaling is involved in multiple cellular processes and is often active in cancer cells, promoting proliferation and metastasis [82]. A_2B_AR was demonstrated to couple to all three types of MAPKs [52], the extracellular signal-regulated kinases (ERK1/2), the stress-activated protein kinases P38 and the c-jun N-terminal kinase (JNK). The cAMP-EPAC pathway and ERK1/2 phosphorylation are known to be involved in A_2B_AR-mediated proliferation of some endothelial cells [55,83]. Limm et al., [61] showed that PKC, but not cAMP or Ca^2+^, is involved in 5′-methylthioadenosine (2)-induced and A_2B_AR-mediated melanoma cell proliferation. Forskolin can mimic adenosine-induced proliferation of MDA-MB-231 breast cancer cells, suggesting that Gs-cAMP signaling is involved, although it is not clear whether PKA or EPAC is the downstream mediator. Recent evidence correlates the A_2B_AR-mediated cAMP/PKA and MAPK/ERK pathway activation with the epithelial-mesenchymal transition in lung cancer cells [49]. A_2B_AR has been shown to activate the PI3K-Akt pathway [52], which is known to induce cell proliferation and protects against apoptosis in many cancer cell types. The A_2B_AR-mediated PI3K-Akt pathway has been shown to be critical for proliferation of glioblastoma stem cells [84]. The importance of Akt signaling in cell survival has been demonstrated in many cell types. However, it remains to be investigated whether the A_2B_AR-mediated PI3K-Akt pathway is downstream of Gαi, Gαs or both.

## 6. A_2B_AR and Tumor Metastasis

A_2B_AR activation plays a critical role in cell motility and migration, which are part of the multi-step process of metastasis [8,56,85]. Adenosine binding to A_2B_AR on tumor cells was found to enhance their metastatic capability [56,86]. It was reported that the A_2B_AR has higher expression in metastatic versus non-metastatic derived colorectal cancer cell lines [21]. A_2B_AR activation has been shown to enhance tumor cell chemotaxis and lung metastasis in animal models of breast cancer and melanoma [5,7,80]. This is consistent with A_2B_AR agonist-induced metastasis, A_2B_AR-selective antagonists and genetic knockdown with shRNA suppressed lung metastasis [5,7,87].

The mechanisms behind A_2B_AR-mediated cell migration and metastasis have been explored [56]. A_2B_AR-mediated cell motility and metastasis is related to the PKA-dependent suppression of Rap1B, a Rho member of the Ras superfamily of small GTPases that activate MAP kinases [56]. It was found that A_2B_AR activation may delay Rap1B prenylation in breast, lung, and pancreatic cancer cell lines, and suggested that A_2B_AR inhibition may be an effective method to prevent metastasis. Similarly, Wilson et al. [88] found that another Gs-coupled GPCR family, the β-adrenergic receptors, suppresses Rap1B prenylation via a PKA-dependent mechanism and promotes the metastatic phenotype in MDA-MB-231 breast cancer cells. Desmet et al. [87] suggested that the enhanced metastasis may involve A_2B_AR-increased gene expression of a key metastatic transcription factor, Fos-related antigen-1 (Fra-1), the expression level of which is associated with increased cell motility and invasion [89,90]. Fra-1 is regulated by ERK, and its overexpression is associated with a poor clinical outcome [91]. Fra-1 and A_2B_AR positively correlate at the mRNA level, and it was shown using chromatin immunoprecipitation (ChIP) experiments that Fra-1 binds the promoter of the A_2B_AR gene in human breast cancer cells [87]. Ou et al. [59] discovered that hypoxia, as well as extracellular ATP, causes a reversible increase in the centrosome-nucleus distance and reduced cell motility through the A_2B_AR and specifically activates the Epac1/RapGef3 pathway. Epac1 is critically involved, and Rap1B is important in the relative positioning of the centrosome and nucleus, which is related to cell motility and migration.

## 7. A_2B_AR and Angiogenesis

Tumor growth is enhanced by angiogenesis, the formation of new blood vessels, which involves the migration, differentiation and growth of endothelial cells inside the blood vessels. Adenosine signaling plays an important role in angiogenesis. Adenosine has been reported to promote angiogenic responses via all four AR subtypes [28,92,93,94]. The endothelial cells express high levels of the A_2B_AR suggesting its potentially critical role in promoting angiogenesis. A_2B_AR stimulation promotes the production of angiogenic cytokines by mast cells [9] and dendritic cells [95]. It has been suggested that adenosine increases endothelial cell proliferation, chemotaxis and capillary tube formation [83,96]. A_2B_AR activation has also been shown to stimulate production of vascular endothelial growth factor (VEGF), basic fibroblast growth factor and insulin-like growth factor-1 (IGF1) by human microvascular endothelial (HMEC)-1 cells [23]. Adenosine was demonstrated to promote VEGF production in rat myocardial myoblasts [97] and in macrophages from C57BL/6 mice [98] It has been demonstrated that AR stimulation could increase VEGF production five-fold in tumor-associated CD45^+^ immune cells, an effect that is not observed in CD45^+^ cells from A_2B_AR KO mice [96] The A_2B_AR induces production of VEGF [23,96,99] and interleukin (IL)-8 in human melanoma cells [46], which are essential for tumor angiogenesis. Bay60-6583, a selective A_2B_AR agonist, was demonstrated to induce in tumor expression of VEGF-A [100]. A_2B_AR inhibition by a selective antagonist PSB-1115 21 significantly decreased tumor growth by blocking angiogenesis and increasing T cells numbers within the tumor microenvironment.

Multiple signaling molecules have been found to be related to A_2B_AR-mediated angiogenesis. Du et al. [101] suggested the A_2B_AR activation-driven angiogenesis is via cAMP-PKA-CREB mediated VEGF production and PI3K/Akt-dependent upregulation of endothelial nitric oxide synthase (eNOS) in HMEC-1 cells. Ryzhov et al. [97] suggested that VEGF appears to be stimulated by a mechanism involving the transcription factor JunB downstream of A_2B_AR-mediated PLC-Rap1-MEK activation. Fang and Olah [55] showed that cyclic AMP-dependent, protein kinase A-independent activation of ERK1/2 following AR stimulation in human umbilical vein endothelial cells was via Epac1.

## 8. A_2B_AR and Immunity

It has been well documented that cancer cells can escape from anti-tumor immune surveillance especially under conditions with impaired immunity. Adenosine has demonstrated its role as an important modulator of immune cell functions at least in part via its action at the A_2B_AR [17,32,41]. A_2B_AR activation is known to suppress IFN-γ-enhanced expression of major histocompatibility complex class II (MHC-II) transactivator [53,102]. In addition to the well-described roles of CD73 and CD39, adenosine deaminase is known to control the local adenosine concentration, and this enzyme also binds to the A_2B_AR [103] Adenosine deaminase deficiency is one of the serious immune diseases which is due to the increased adenosine concentration and subsequently suppressed immune responses. Thus, in addition to its direct effects on metastasis, proliferation and angiogenesis, the A_2B_AR can have a direct or an indirect role on cancer progression via modulation of the immune system. The role of the A_2B_AR in cell immunity was mostly neglected until recently partly due to adenosine having a low A_2B_AR affinity [11,12], although early findings indicated that A_2B_AR was the AR subtype responsible for the immune suppressive function of T cells, macrophages and dendritic cells [11,17,41]. Also, early work on CD26/DPP4 (dipeptidyl peptidase 4), a T cell surface antigen that cleaves various bioactive peptides, mainly focused on its role in T cells [104,105] that highly express the A_2A_AR [106,107,108]. More recently, in addition to CD39 and CD73, the importance of A_2B_AR and DPP4 in dendritic cells and macrophages also gained appreciation [47]. DPP4 has been identified as one of the macrophage-related gene signatures predicative of increased risk in gliomas [109]. DPP4 inhibitor vildagliptin has been reported to suppress lung cancer growth via a macrophage-mediated mechanism [110]. Considering the increased adenosine concentration and increased A_2B_AR expression in the tumor microenvironment [17,31,100] together with the high expression levels of both A_2B_AR and DPP4 in macrophages and dendritic cells, growing evidence suggests a critical role of A_2B_AR together with CD39 and CD73 in modulating cancer progression at least in part via immune suppression. Furthermore, DPP4 physically associates with adenosine deaminase, which controls adenosine concentration and binds to the A_2B_AR. Thus, A_2B_AR blockade may enhance the function of immune cells [17,31,41].

A_2A_AR has been shown to be critical in regulating toll-like receptor (TLR)-induced cytokine production. However, a recent study utilizing macrophages isolated from A_2B_AR KO mice showed that adenosine elicits IL-6 production from macrophages via the A_2B_AR [19]. IFN-γ upregulates A_2B_AR expression on macrophages resulting in an increased responsiveness of macrophages to the stimulatory effects of NECA [111]. The pharmacologic inhibition or the genetic deletion of the A_2B_AR results in a hyperinflammatory response to TLR ligation, similar to IFN-γ treatment of macrophages, suggesting the NECA-mediated effect is via A_2B_AR, but not A_2A_AR [111]. The role of A_2B_AR in regulating dendritic cell function has been defined using A_2B_AR KO mice and selective agonists and antagonists for A_2B_AR [17,41]. In mice bearing MB49 and/or 4T1 tumors, Cekic et al. [5] demonstrated that selective blockade of A_2B_AR resulted in a C-X-C chemokine receptor 3 (CXCR3)-dependent reduction of tumor growth and lung metastases from breast tumors through enhancement of dendritic cell activation. Inhibition of A_2B_AR activation by PSB-603 was shown to suppress regulatory T cell (Treg) differentiation and IL-10 production, without affecting effector T cell activation measured by IL-2 production and CD25 expression [112]. A_2B_AR was also suggested to modulate the phenotype of bone marrow-derived dendritic cells. A_2B_AR activation impairs MHC-II transcription in IFN-γ-stimulated cells [113,114]. MHC-II expression is required for CD4^+^ T cell anti-tumor responses, and loss of MHC-II is associated with aggressiveness of colorectal cancer and decreased levels of tumor-infiltrating lymphocytes [115]. Shi et al. [116] also reported that both major MHC-II transactivator (CIITA) and MHC-II are decreased in highly metastatic cancer cells. Thus, A_2B_AR blockade has the potential to enhance anti-tumor immunity in cancers where tumor-infiltrating lymphocytes and MHC-II levels are decreased.

The specific signaling pathways related to A_2B_AR-mediated immune suppression have been explored. Xu et al. [53] found that A_2B_AR-mediated cAMP is both necessary and sufficient to suppress the IFN-γ-mediated immune response. Figueiredo et al. [117] showed that cAMP accumulation induced by A_2B_AR activation is important to inhibit dendritic cell activation and to evade the immune response in infected mice. In human monocytes, it has been suggested that A_2B_AR-triggered cAMP accumulation inhibits the immune response by lowering the amount of MHC class I and class II molecules [118]. A_2B_AR-induced cAMP accumulation was also found to reduce STAT1 phosphorylation and impair its binding to the CIITA promoter while fostering synthesis of TGF-β, known to antagonize MHC-II transactivation [113,114]. Iannone et al. [10] showed that melanoma-bearing mice treated with the selective A_2B_AR agonist BAY60-6583 had increased melanoma growth, which was associated with higher levels of immune regulatory mediators IL-10 and monocyte chemoattractant protein 1 and accumulation of tumor-associated CD11b+ and Gr1+ cells and myeloid-derived suppressor cells. Depletion of CD11b+Gr1+ cells completely reversed the pro-tumor activity of BAY60-6583. Inhibition of A_2B_AR with PSB-1115 reversed immune suppression in the tumor microenvironment, leading to a significant delay in melanoma growth. The authors suggest that the antitumor activity of PSB-1115 relies on its ability to lower accumulation of tumor-infiltrating myeloid-derived suppressor cells (MDSCs) and restore an efficient antitumor T cell response.

## 9. A_2B_AR Antagonists as Novel Anticancer Agents

As described above, A_2B_AR activation induces tumor proliferation, growth of solid tumor, tumor angiogenesis, tumor cell invasion and metastasis, and immune suppression. Thus, A_2B_AR blockade holds great promise as an anti-cancer therapy. For example, A_2B_AR inhibition by the antagonist PSB-1115 was shown to decrease tumor metastasis of CD73^+^ melanoma cells and mammary carcinoma cells [7] Iannone et al. [10] observed that PSB-1115 delayed tumor growth and enhanced the anti-tumor activity of dacarzabine, a drug currently used in metastatic melanoma.

Cekic et al. [5] demonstrated that the antitumor effect of theophylline occurs via the A_2B_AR rather A_2A_AR, based on a study using A_2A_ and A_2B_AR KO mice. Nevertheless, simultaneous antagonism of both subtypes has been proposed to be possibly synergistic against some types of tumors [17,32], although it is not clear whether the blockade of both A_2A_AR and A_2B_AR could also produce more adverse effects than either subtype separately.

Antagonists in clinical trials for cancer patients (ClinicalTrials.gov NCT Identifier) include the mixed A_2A_AR/A_2B_AR antagonist AB928 26 (Phase 1, lung cancer, 03846310; Phase 1, breast and ovarian cancer, 03719326; Phase 1, gastrointestinal cancer, 03720678; Phase 1, advanced cancer, 03629756), PBF-1129 (structure not disclosed; Phase 1, non-small cell lung cancer, 03274479) and theophylline 11 (see below). The first dual-acting A_2A_AR/A_2B_AR antagonist AB928 is being tested clinically in multiple arms in combination with pegylated liposomal doxorubicin, nanoparticle albumin-bound paclitaxel, or a PI3K-γ inhibitor. AB928 has exhibited excellent safety, PK, and PD profiles in a Phase 1 clinical trial in healthy volunteers and is currently being evaluated in patients with non-small cell lung cancer, breast cancer, ovarian cancer, colorectal and six other types of cancers (clinicaltrials.gov). One of the cancer immunotherapy drugs, AB122, a fully human immunoglobulin G4 monoclonal antibody targeting human programmed cell death protein 1 (PD-1), will be tried in combination with AB928. AB928 was able to produce maximal AR blockade assessed as a function of NECA-stimulated pCREB induction in peripheral blood CD8+ T cells [3]. AB928 was shown to relieve adenosine-mediated immune suppression [76]. Combining AR inhibition with AB928 and chemotherapy results in greater immune activation and tumor control.

A phase I trial of the selective A_2B_AR antagonist PBF-1129 (structure not disclosed) in patients with advanced non-small cell lung cancer is being conducted. PBF-1129 is being administered in a dose escalation study of tolerability without other therapy.

Theophylline is a nonselective AR antagonist, which was tested for anticancer efficacy in two previous clinical trials (incidentally, as an inhibitor of intracellular cAMP in chronic lymphocytic leukemia, Phase 2, 00003808; a withdrawn trial in combination with an allogeneic tumor cell-vaccine (gp96-Ig vaccine) and oxygen therapy, which lowers adenosine levels [119], in non-small cell lung cancer, Phase 1, 01799161). The first theophylline trial description did not even reference AR antagonism, but there was a correlation found between in vitro apoptosis in leukemia cells and clinical response in a subset of patients [120]. Theophylline in combination prednisone and dextromethorphan has also been in a clinical trial (Phase 1, 01017939) for patients with metastatic castration-resistant prostate cancer. Aminophylline, a salt of theophylline, in combination with Bacillus Calmette-Guerin has been in a trial (early Phase 1, 01240824) for patients with bladder cancer. However, it should be noted that theophylline is nonselective and may block all four ARs.

## 10. Summary

A_2B_AR signaling is a major pathway contributing to cancer cell proliferation and solid tumor growth, angiogenesis and metastasis, and immune suppression. Thus, A_2B_AR antagonists are potentially a novel anticancer therapy, either in combination with other anticancer drugs or as a mono-therapy. Several A_2B_AR antagonists are now in clinical trials for patients with various types of cancers. The nonselective A_2B_AR antagonist, theophylline, in combination with other anticancer drugs has been evaluated in patients with bladder cancer and prostate cancer. Dual acting A_2A_AR/A_2B_AR antagonist AB928 has exhibited excellent safety, PK, and PD profiles in a Phase 1 clinical trial in healthy volunteers and is currently being evaluated in patients with non-small cell lung cancer, breast cancer and ovarian cancer. A_2B_AR selective antagonist PBF-1129 is also in clinical trial for patients with non-small cell lung cancer. Thus, A_2B_AR antagonism is a promising direction for the development of new cancer therapeutics.

## Figures and Tables

**Table 1:** Binding affinity (K_i_, nM) or functional potency (EC_50_, nM; E_max_ as %) of commercially available A_2B_AR agonists and antagonists as pharmacological tools and A_2B_AR antagonists in clinical trials for cancer patients. Refer to Figure 2 for structures. K_i_ (nM) or EC_50_ (E_max_, %)

Compound	A_1_	A_2A_	A_2B_	A_3_	Reference
*Agonists*					
1, Adenosine ^a^	310	700	24,000	290	[68]
			4620 ^c^ (97%)		[37]
3, NECA ^b^	14	20	1900	25	[38]
3, NECA ^a^	12	60	104 (100%)	11	[71]
4, CPCA	1.9^b^	50^b^	267 ^c^ (102%)	108 ^b^	[37]
6, BAY68-4986 ^a^	0.66	1400	1.1 (93%)	2400	[70]
(Capadenoson)			522 ^c,d^ (95%)		
7, LUF5834	2.6 ^b^	28 ^b^	12 ^a^ (74%)	538 ^b^	[71]
8, BAY60-6583 ^b^	390	>10,000	110	220	[38]
			242 ^c^ (73%)		[37]
			6.1 ^e^ (102%)		[37]
*Antagonists*					
11, Theophylline ^b^	6200	1710	7850	22,300	
12, Caffeine ^b^	44,900	9560	33,800	13,300	[38]
13, MRS1754 ^b^	403	503	1.7	570	[38]
14, MRS1706 ^b^	157	112	1.4	230	[38]
18, GS6201 ^b^ (CVT-6833)	1940	3280	22	1070	[74]
21, PSB-1115 ^b^	>10,000	3790	53.4	>10,000	[38]
22a, PSB-603 ^b^	>10,000	>10,000	0.55	>1000	[38]
22b, PSB-1901 ^b^	>1000	>1000	0.060	>1000	[73]
23, PSB-0788 ^b^	2240	333	0.39	>1000	[38]
27, LAS101057 ^b^	>10,000	2500	24	>10,000	[75]
26, AB928 ^b^	64	1.5	2.0	489	[76]
27, ISAM140^b^	>10,000	>10,000	0.55	>1000	[77]
PBF-1129	nd	nd	nd	nd	

^a^ EC_50_ values (nM) from cAMP assays. ^b^ K_i_ values (nM) from radioligand binding. ^c^ EC_50_ values (nM) from cAMP assays in human embryonic kidney (HEK)293 cells endogenously expressing the A_2B_AR. ^d^ unpublished data. ^e^ The EC_50_ and E_max_ values of Bay60-6583 stimulated cAMP accumulation in HEK293 cells expressing the recombinant human A_2B_AR [37]; Percentages shown in the A_2B_ column represent the agonist E_max_ in comparison to NECA as 100%. nd, not disclosed.
